# Ecophysiological Responses of the Lesser Mealworm *Alphitobius diaperinus* Exposed to Desiccating Conditions

**DOI:** 10.3389/fphys.2022.826458

**Published:** 2022-02-23

**Authors:** Julie Engell Dahl, David Renault

**Affiliations:** ^1^Université de Rennes, CNRS, EcoBio (Ecosystèmes, Biodiversité, Évolution)—UMR 6553, Rennes, France; ^2^Department of Bioscience, Aarhus University, Aarhus, Denmark; ^3^Institut Universitaire de France, Paris, France

**Keywords:** beetle, survival, body mass, metabolites, GC-MS, sugar, activity, reproduction

## Abstract

In order to improve predictions of the impacts of climate change on insects, this study aimed to uncover how exposure to dry conditions can affect the biology of the invasive pest beetle *Alphitobius diaperinus* in terms of longevity, activity, water content, metabolic profiles, and fecundity. We measured desiccation resistance in adults of *A. diaperinus* by recording the time the beetles could survive desiccation stress. We found that the species was highly desiccation resistant, with about 50% of the insects exposed to desiccation being able to survive for 30 days, and some individuals even survived for up to 50 days at 10% ± 2 relative humidity. There was no evidence of active upregulation of sugars or other metabolites which the beetles could have used to better tolerate desiccation. Food deprivation affected both control (food deprivation, no desiccation) and treatment (food deprivation, desiccation) groups, as their metabolic phenotypes changed similarly after 1 week of treatment. Also, the activity of beetles from both control and desiccation treatments was similarly increased 2 weeks after the experiment had started. Even if there were no changes in the metabolic phenotypes of the insects experiencing desiccating conditions, beetles exposed to desiccation for 8 days had a significantly reduced reproductive output as compared with control insects. This result indicated a physiological cost of drought resistance or repair of stress-incurred damages. The exact nature of that effect (e.g., direct or indirect physiological costs) has not yet been described for tenebrionid beetles and should be investigated in future studies.

## Introduction

Changes in the frequency and/or intensity of dry spells and heat waves threaten terrestrial biodiversity by increasing the likelihood of individuals’ desiccation (Trenberth, 2011; IPCC, 2014). This is particularly true for smaller animals such as insects which have a large surface-to-volume ratio and are thus highly susceptible to evapotranspiration and dehydration (Hadley, 1994). Therefore, they have developed a wealth of behavioral, morphological, and physiological adaptations aiming at limiting loss of body water and/or detrimental consequences thereof (Hadley, 1994; Hidalgo et al., 2014, 2018; Mamai et al., 2014, 2016; Thorat and Nath, 2018). Knowledge from investigating the nature of these adaptations, and the variation in such traits within and across species, can contribute to improving predictions of the current and future impacts of climate change (Helmuth et al., 2005; Chown and Gaston, 2008).

Behavioral responses can be critically important for the survival of insects facing changes in humidity conditions (Rowley and Hanson, 2007). If possible, insects will avoid dry microhabitats and relocate toward more humid environments, as shown in the yellow-spined bamboo locust *Ceracris kiangsu* (Yu et al., 2010), as well as in several other species (Heger et al., 2006; Rowley and Hanson, 2007; Kessler and Guerin, 2008). If a more optimal microhabitat is not available, or if the distance to reach it is too high, insects must then rely on morphological and physiological adaptations to survive stressful dry spells. In insects, the main physiological responses to desiccation include (i) limiting the rate of body water loss (desiccation resistance) (Ahearn, 1970; Zachariassen, 1991), (ii) increasing tolerance to water loss (desiccation tolerance) (Arlian and Staiger, 1978; Hadley, 1994), and (iii) controlling the main routes of water loss (excretion, transpiration, and respiratory loss) (Chown et al., 2011).

For many desert beetles, cuticle permeability represents a prominent source of body water loss, and adjustment in cuticle composition can thus greatly contribute to enhancing desiccation resistance (Ahearn, 1970; Cloudsley-Thompson, 2001). Other studies have shown that the tenebrionid sub-elytral cavity can also effectively reduce transpiration (Ahearn and Hadley, 1969; Draney, 1993), further increasing the level of desiccation resistance of the insects. Activity reduction (for instance foraging, reproduction, food intake) decreases respiratory metabolism, and thus lowers transpiration, and this also enhances the capabilities of insects to handle dryer environmental conditions (Williams et al., 1998). Consistently, a study comparing *Drosophila* flies from desert and mesic regions showed that species from dry regions mainly increased resistance to desiccating conditions by reducing activity and metabolic rate as compared with mesic species, thereby decreasing respiratory water loss (Gibbs et al., 2003).

Desiccation resistance or tolerance can also be enhanced by modulation of hemolymph osmolarity, by for instance accumulation of sugars and polyols in the insects’ body fluids (Gray and Bradley, 2005). In terms of desiccation tolerance, trehalose has been reported as an important metabolite for insects subjected to harsh desiccating conditions and is even indispensable for those invertebrates capable of anhydrobiosis (Crowe, 2002; Watanabe et al., 2002; Mensink et al., 2017; Thorat and Nath, 2018). Trehalose helps to maintain the integrity of cell membranes and proteins (Kikawada et al., 2007). Interestingly, the level of several amino acids, sugars, and sugar alcohols typically increases in response to different stress conditions (Guy et al., 2008; Colinet et al., 2012; Krasensky et al., 2012), in particular when osmolality of the body fluids and electrochemical gradients are altered by the stressors. As a result, the accumulation of these low molecular weight compounds (often referred to as compatible osmolytes) is also expected to help cells to reduce water loss during desiccation (Yancey, 2005). In addition to helping cells in maintaining osmotic pressure, compatible osmolytes may have other protective functions, such as protein (Mensink et al., 2017) and membrane (Crowe, 2002) stabilization, and may have antioxidative properties (Liang et al., 2013). Therefore, describing changes in metabolite composition in insects during stressful conditions can improve our knowledge of the insect coping strategy.

It is generally admitted that reproduction entails a cost, and stress susceptibility in insects could partly result from this reproduction cost (Wang et al., 2001; Salmon et al., 2007). On the other side, stress and/or repeated stress exposure may also result in a reproduction-survival trade-off (Marshall and Sinclair, 2010), as resources can be reallocated from reproduction toward other biological processes and physiological functions to improve the overall individual’s fitness (Chown and Nicholson, 2004). Consistently, increased stress tolerance has often been negatively correlated with life-history traits other than longevity *in Drosophila* (Hoffmann and Parsons, 1989). Moreover, when coping with stress, reproductive fitness can also be reduced by the alteration of the quality of gametes or progeny (Schreck et al., 2001). While some studies have found negative associations between dry conditions and reproduction in insects in the field (Vessby, 2001; Yaro et al., 2012), there is a scarcity of knowledge on how desiccation could affect their reproduction.

In the present study, we investigated the ecophysiological responses of the tenebrionid beetle, *Alphitobius diaperinus* Panzer exposed to desiccating conditions. This insect of tropical origin has invaded Europe and nowadays represents one of the most abundant pest insects in poultry houses (Rueda and Axtell, 1997). Owing to its origin and former investigations on the species, the beetle is expected to have developed high capacities to handle desiccation exposures (Zachariassen and Einarson, 1993; Renault and Coray, 2004; Renault et al., 2015). Our aim was to uncover how exposure to dry conditions can affect the biology of this beetle in terms of longevity, activity, water content, metabolic phenotypes, and fecundity. We measured the responses to desiccation exposure in adults of *A. diaperinus* by recording the time the beetles could survive desiccation and hypothesized that stress exposed insects would be characterized by a shorter duration of survival relative to controls. We also hypothesized that animals exposed to the desiccating condition would lose body water content over time, and not control insects, and that insects exposed to desiccation would be characterized by increased internal concentration of soluble sugars (osmolytes). Furthermore, we expected the activity of stressed beetles to be lower, thereby reducing respiratory water loss. Finally, in order to measure any effect of desiccation on fecundity, we determined the number of offspring after the insects were exposed to desiccating conditions for 8 days. We expected animals having experienced desiccation to produce fewer offspring than control ones.

## Materials and Methods

### Collection and Rearing of Insects

Adults of *A. diaperinus* were hand-collected from a poultry house at Miniac-sous-Bécherel (Brittany, France; 48°17′10″ N, 1°55′51″ W) in October 2019. After collection, the insects were transported to the laboratory (UMR EcoBio, University of Rennes, Rennes, France) and kept in plastic containers (30 × 30 × 13 cm, L × l × h) in the dark at constant 25 ± 1°C (Lovidond incubators, TC 225 S, Tintometer GmbH, Sarasota, United States). The insects were fed *ad libitum* with pellets of dry dog food and oat, and supplied with tap water in 2 mL microtubes closed by wet cotton. The age and sex of the insects were not controlled for the experiments as we were aiming at getting estimates of the responses of the insects to desiccating conditions that would be representative of the responses at the populational scale.

### Effects of Desiccating Conditions on the Survival and Physiology of the Insects

#### Survival of the Insects Exposed to Desiccating Conditions

To measure the survival capacities of adult *A. diaperinus* continuously exposed to desiccating conditions and food deprivation, in comparison to controls (non-desiccating conditions, food deprivation), groups of 10 beetles were transferred to 24 round plastic containers (5 cm diameter, 4 cm tall), without food. Four containers were placed into each of three distinct larger and transparent plastic boxes (35 × 25 × 10 cm), whose bottom was covered with 3 cm of desiccant (silica gel). The plastic boxes were hermetically closed. Three additional plastic boxes (35 × 25 × 10 cm), each again containing four round containers of 10 beetles, were settled and served as controls (wetted cotton balls were placed at the bottom of the plastic boxes to maintain relative humidity (RH) optimal). All insects were deprived of food during the whole duration of the experiment to avoid survival differences that would have inevitably occurred among stressed insects progressively falling into coma, thus preventing them from feeding, and control insects that were assumed to remain active. In each container, the groups of 10 beetles were scored for survival twice a week for 6 weeks at room temperature (22 ± 2°C). At each observation, the beetles were categorized as alive (walking and fit beetles), or dead (insects without visible movement of legs or other body appendage, even when the plastic boxes were gently knocked to induce vibrations that stimulated behavioral responses of alive insects). In the boxes, RH and temperature were monitored with Hygrochron ibuttons^®^ (Mouser electronics, Brive La Gaillarde, France). Over the course of the experiment, RH was 10 ± 2 and 60 ± 5% in desiccation and control treatments, respectively.

#### Measurements of Body Water and Sugar Contents

Changes in body water and sugar contents were monitored by setting two additional plastic boxes (35 × 25 × 10 cm) for each experimental condition (no desiccation, desiccation). Here, five containers, each containing groups of 20 insects, were set up in each plastic box. Once every week, eight replicates of three individuals were taken from each treatment (no desiccation, desiccation). The fresh mass of each pool of three insects was measured directly after collection, and the samples were then transferred in a dryer (60°C) for 3–4 days, before dry mass was measured. Water content was calculated as the difference between fresh and dry masses for each replicate of three individuals, in relation to their respective fresh mass. After 5 weeks of the experiment, the number of survivors was drastically reduced in the desiccation treatment, and only three replicates of three insects were made for the determination of water and sugar contents.

Total soluble sugars were measured from the samples whose fresh and dry masses were determined (six replicates of three insects for each experimental condition, except for the insects exposed for 5 weeks to desiccating condition); we used the method described by Foray et al. (2012). Briefly, two tungsten beads (Ø = 3 mm) were added to each sample before they were homogenized in 750 μL of a 2:1 (v:v) mixture of ice-cold methanol:chloroform with a bead beater (Retsch) at 25 Hz for 1 min 30. Then, a volume of 500 μL of ice-cold ultrapure water was added to each sample, and they were vortexed before being centrifuged at 4,000 g for 10 min at 4°C. Four hundred microliter of the upper alcoholic phase of the supernatant was pipetted, and let dry for 30 min under a fume hood. The remaining 600 μL of the methanol:H_2_O solution was stored at –25°C before being used for the subsequent metabolomics analyses (see section “Metabolic Profiles”). Total soluble sugars were measured by adding 1,000 μL of a solution of Anthrone mixed in 70% H_2_SO_4_. The samples were plunged in a water bath at 90°C for 15 min. The absorbance of the samples was then determined using a spectrophotometer at 625 nm. A calibration curve was made using known concentrations of glucose, and this curve was used for the quantification of the content in soluble sugars in the samples based on their respective absorbance.

#### Metabolic Profiles

The GC-MS analyses were performed by using the alcoholic extracts prepared from sugar measurements (see section “Measurements of Body Water and Sugar Contents”). From the remaining 600 μL of the methanol: H_2_O extract, an aliquot of 300 μL was transferred to new glass vials. Samples were vacuum dried (Speed Vac Concentrator, MiVac, Genevac Ltd., Ipswich, England). We used the derivatization process described by Khodayari et al. (2013), which relies on the use of a CTC CombiPAL autosampler (CTC Analytics AG, Zwingen, Switzerland) ensuring on-time preparation and analysis of the samples. The analyses were performed with a GC-MS platform comprising an Agilent 7890B gas chromatograph coupled to a 5977B mass spectrometer. We used the same settings as those described in Thiébaut et al. (2021). The detected peaks (electron energy: 70 eV; in full scan mode) were annotated with MassHunter. Concentration of each metabolite was calculated using individual quadratic calibration curves.

### Effects of Desiccating Conditions on Activity and Reproductive Capacities of the Insects

#### Measurement of Locomotor Activity

For each experimental treatment (no desiccation, desiccation), the activity of individual beetle was measured once at the start of the experiment and after 2 weeks of the experiment. Petri dishes whose bottom was covered with squared paper (1 cm^2^) were used to measure beetle’s activity, which was recorded as the number of times the beetle crossed a line during 1 min. The locomotor activity of 30 individuals was assayed for each treatment (no desiccation, desiccation); the tested insects were collected from the plastic boxes settled for the determination of water and sugar contents.

#### Measurement of the Reproduction Capacities

We assessed the long-lasting effects desiccating conditions could have on the fitness of the beetles by measuring their subsequent reproductive capacities. To that aim, two sets of seven plastic boxes (30 × 25 × 10 cm) were set up for controls and desiccation treatment, as described in the survival experiment section. Six small plastic containers (5 cm diameter, 4 cm tall), each containing 15 insects, were placed in each plastic box for 8 days at 25°C. In this experiment, RH measured from the desiccation boxes was 14% RH ± 2% (RH measurements recorded every second hour with Hygrochron ibuttons^®^, Mouser electronics, Brive La Gaillarde, France). As the duration of the exposure would not induce a comatose state in any insects, and as we were interested in testing the impact of desiccation on reproductive capacities of the beetles, food in the form of dog food pellet was added in the plastic containers; control containers were all additionally provided with a microtube with water and cotton for *ad libitum* water access. After 8 days of experimental treatment, plastic boxes were briefly opened to assess the health status of the individuals; no mortality (mortality was defined as no movement and lack of behavioral responses of the insects when stimulated) was observed. Then, groups of 50 insects (sex ratio about 50:50) from the same treatment were transferred to new plastic boxes (15 × 25 × 10 cm) with lids that had an approximately 10 cm^2^ aeration hole. The bottom of the plastic boxes was covered with approximately 0.5 cm of fine wheat flour, topped with 0.7 cm of organic oat bran. A piece of organic carrot weighing approximately 8 g was placed in each container as a source of water for adults (there was no microtube with water provided to the insects here, as females might have laid eggs on the piece of cotton), and to provide a moist area under which the adults could lay their eggs. After 1 week in these boxes, adults were removed by sieving the food. The food and pieces of carrots were then returned to their respective boxes so that eggs, and later larvae, could develop and grow. Thirty days after the removal of the adults (food and water were refilled whenever necessary to ensure *ad libitum* access), food was sieved to get the larvae separated from the food, and larvae were counted.

### Statistics

All statistical tests were run in R version 3.5.2. Difference in survival probability of the insects between control (no desiccation, ND) and desiccation (D) treatment was compared using Kaplan Meier plot with a log-rank test, using the survminer package in R. Changes in water and sugar contents over time were measured using a two-way ANOVA, with *post hoc* multiple comparisons using multiple *t*-tests and Bonferroni adjusted *P*-values. The effects of the experimental treatments (no desiccation, desiccation) on the metabolic phenotypes of the insects were investigated using Principal Component Analysis (PCA). Multiple correlation coefficient (*R*^2^) and cross-validated *R*^2^ (*Q*^2^) were used to confirm the predictive power of the fitted model, and the statistical significance of the PCA was also assessed with permutation tests (1,000 permutations, *P* <0.001). Loadings of the PCA components are presented in Supplementary Appendix 1. The analysis was computed with the MetaboAnalyst platform on cube root-transformed metabolite concentrations that were additionally auto-scaled. Original and transformed metabolite concentrations are presented in Supplementary Appendix 2. Osmolarity of the body fluids of the insects, based on the total quantity of quantified metabolites, was calculated and compared using a two-way ANOVA with type III sum of squares, as the number of samples in each group was not always identical. Differences in activity and number of offspring in insects from control and desiccation treatments were tested using one-way ANOVAs with pairwise comparison using *t*-tests for the activity measures^1^.

## Results

### Survival of the Insects Exposed to Desiccating Conditions

**FIGURE 1:**
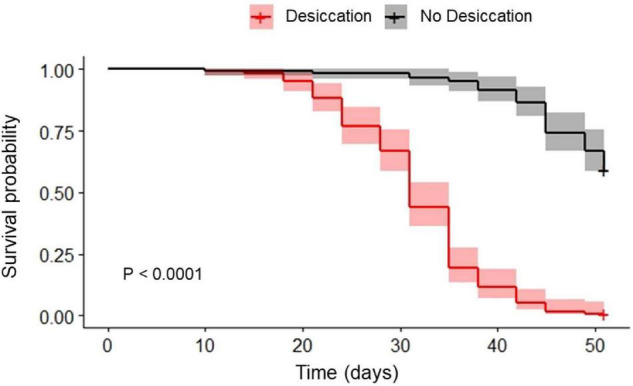
Survival probability of food-deprived adults of *Alphitobius diaperinus* kept at 10 ± 2% RH (desiccation, gray line) and their relatives kept at approximately 60% RH (no desiccation, black line). *P*-value resulting from log-rank test comparing the two Kaplan Meier plots is presented, as well as the 95% confidence intervals for each survival curve. “+” at the end of the survival curves indicate that data were censored.

Desiccation significantly reduced the survival probability of the beetles (*P* < 0.001), with about 50% of the individuals being dead after 30 days of the experiment, while almost no mortality was recorded in the controls (ND) after 30 d (Figure 1). All insects from the desiccating condition were dead at day 50, while approximatively 60% of control beetles were still alive at that time.

### Effects of Desiccating Conditions on Water and Sugar Contents and Metabolic Profiles

**FIGURE 2:**
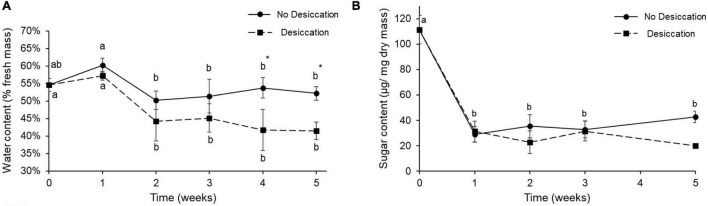
Water content (mean ± SD) **(A)** and sugar content (Mean ± SD) **(B)** of food-deprived insects kept at 10 ± 2% RH (desiccation treatment, dotted line and black squares) and control insects kept at 60% RH (no desiccation, solid line, and black dots). For each treatment, letters denote significant differences over the duration of the experiment. Stars (*) indicate significant differences between the two treatments at each time point (two-way ANOVA).

After two weeks of the experiment, the water content of the beetles maintained under desiccating conditions was significantly reduced, and was significantly different than in control beetles at week 4 (*P* < 0.05) (Figure 2A). Body water content of control beetles remained relatively stable over the course of the experiment (Figure 2A). The body sugar content was drastically reduced 1 week after the experiment had started, and then remained stable—at level around 20 μg/mg dry mass for the beetles of both treatments (Figure 2B).

**FIGURE 3:**
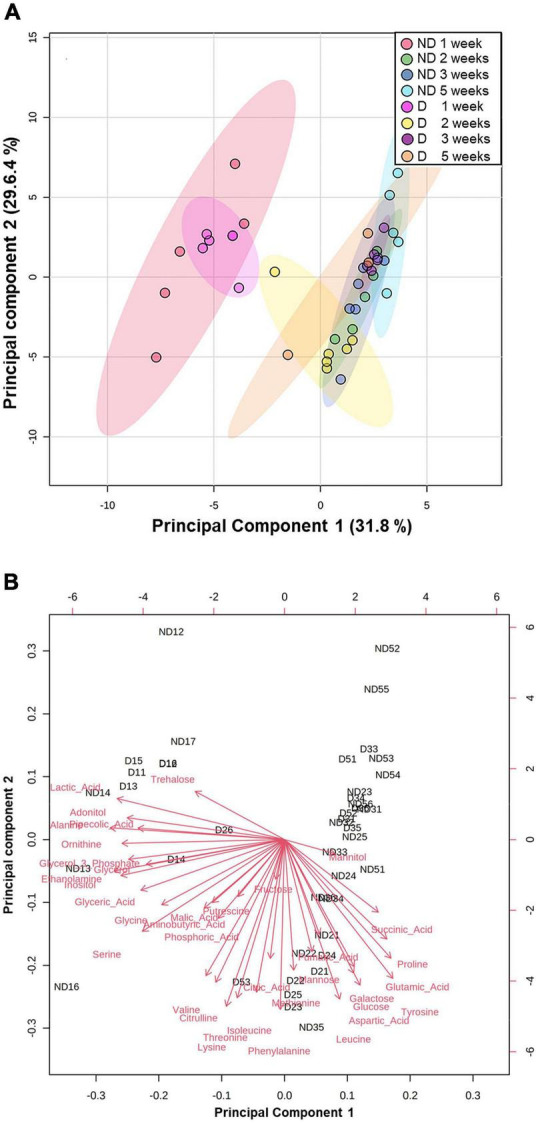
Principal component analysis (PCA) score plots of metabolites data from adult *Alphitobius diaperinus* on the first two principal components (PC1 and PC2) **(A)** PCA of metabolite data from food-deprived beetles exposed at 60% RH (No Desiccation, ND) or 10% RH (Desiccation, D) for 1, 2, 3, or 5 weeks. Colored dots represent individual samples. Colored ovals represent 95% confidence intervals. Percentages of total variance of PC1 and PC2 are presented in parentheses. **(B)** Biplot of the PCA. Red arrows represent individual metabolite loading vector; the magnitude (distance from the origin) and direction of the loading vectors demonstrate the contribution of each metabolite to sample clustering. The loading plots graphing the coefficients of each metabolite for the first and second components of the PCA are presented in Supplementary Appendix 1. Samples Id read as follows: ND = beetles exposed at 60% RH; D = beetles exposed at 10% RH. The first number refers to the duration of exposure (in weeks); the second number corresponds to the numbering of the samples. For example, ND35 means the 5th replicate of the beetles that were exposed at 60% RH for 3 weeks.

**FIGURE 4:**
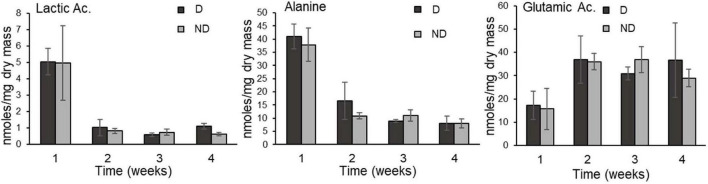
Lactic Acid, Alanine, and Glutamic Acid (mean ± SD) in food-deprived adults of *Alphitobius diaperinus* exposed at 10 ± 2% RH (desiccation treatment, D) or at 60% RH (no desiccation, ND); there was no consistent difference between the two treatments over the duration of the experiment. Contents of all metabolites quantified by GC-MS are presented in Supplementary Appendix 2.

The GC-MS analysis allowed us to accurately detect and quantify 37 metabolites (Supplementary Appendix 2). The resulting metabolic profiles were analyzed with a principal component analysis (PCA) to assess changes in the concentrations of individual metabolites by experimental group (Figures 3A,B). The first two dimensions (Principal component 1: PC1; Principal component 2: PC2) of the PCA captured 61.4% of the total inertia in the dataset, with PC1 and PC2 accounting for 31.8 and 29.6% of the total inertia, respectively (Figure 3A). PC1 mainly discriminated two groups, with the beetles exposed to ND and D conditions for 1 week clustering together and differentiating from the other experimental treatments which had a tendency to group together. The metabolites that contributed the most to PC1, and that were recorded in higher amounts in adult *A. diaperinus* exposed to ND and D conditions for 1 week were Alanine, Ethanolamine, Lactic Acid, Inositol, Ornithine, Adonitol and Glycerol 3 Phosphate (Figures 3B, 4 and Supplementary Appendices 1, 2). Conversely, beetles from the other treatment groups had higher concentrations of Tyrosine, Glutamic Acid, Proline, Succinic Acid, Aspartic Acid, Galactose, and Glucose (Figures 3B, 4 and Supplementary Appendices 1, 2).

**FIGURE 5:**
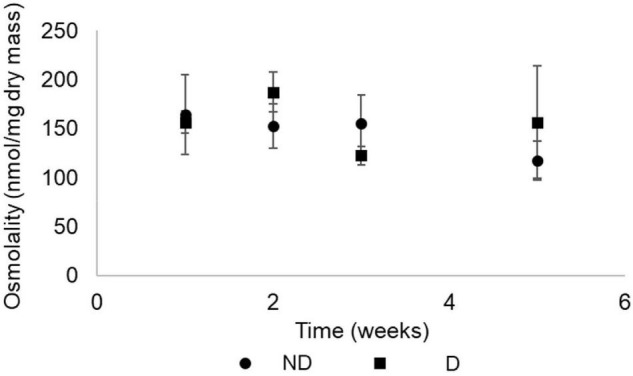
Osmolarity (Mean ± S.D.) of food-deprived adults of *Alphitobius diaperinus* exposed at 10 ± 2% RH (desiccation treatment, D) or 60% RH (no desiccation, ND). Osmolarity was calculated by summing the quantities of all metabolites that were measured by GC-MS and expressed as nmoles/mg dry mass. A two-way ANOVA (using type III sum of squares) showed no effect of time (*p* = 0.063) or treatment (*p* = 0.669).

Osmolarity, calculated from the quantities of measured metabolites from the body fluids of the insects, remained constant over the experimental period for both treatments (Figure 5).

### Locomotor Activity of the Beetles

**FIGURE 6:**
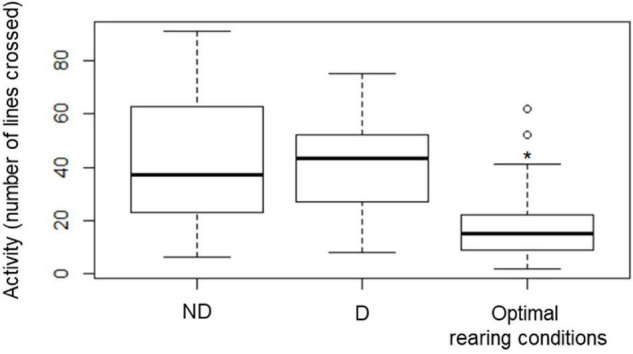
Activity of food-deprived (starved) insects kept at 60% RH (no desiccation, ND) or 10 ± 2% RH (desiccation, D) for 2 weeks. The assay was also conducted at the start of the experiment, allowing us to obtain an estimate of the activity of the beetles reared under optimal laboratory conditions. Activity was measured as the number of times an individual crossed a line in a Petri Dish whose bottom was covered with a line grid. The boxplots present median (horizontal line), 25th and 75 percentiles (box), and the standard deviation (error bars) of the recorded activity. Star (*) indicates significant difference from the two other groups, as revealed by pairwise *t*-tests (*P* < 0.001) following a one-way ANOVA.

The activity of the beetles increased significantly after 2 weeks of the experiment (*P* < 0.001) as compared with the activity of the insects measured at the start of the experiment (Optimal rearing conditions). After 2 weeks, activity was similar in insects from the two experimental treatments (ND, D) (Figure 6).

### Reproductive Capacities

**FIGURE 7:**
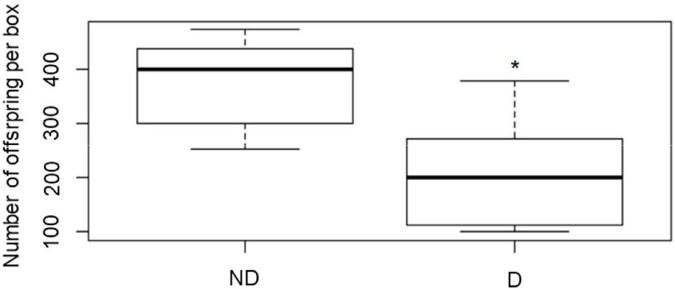
Number of offspring recorded in insects after they were kept for 8 days at 60 (no desiccation, ND) or 14 (desiccation, D) % RH, before being returned to optimal rearing conditions for 1 week, followed by 30 days of monitoring of the development of the offspring. The boxplots present median (horizontal line), 25th and 75 percentiles (box), and the standard deviation (error bars). * Indicates significant difference shown by a one-way ANOVA (*P* < 0.001).

Insects having experienced 8 days of desiccation had significantly fewer offspring as compared with their control relatives (*P* < 0.001) (Figure 7).

## Discussion

Desiccation resistance or tolerance greatly contribute to shaping the geographic and habitat distribution of arthropod species (Dias et al., 2013; Ouisse et al., 2016). The different strategies that terrestrial arthropods have evolved for coping with desiccating conditions include increased body water amounts, reducing body water losses, or tolerating high levels of dehydration (Hadley, 1994; Hidalgo et al., 2014, 2016). While improved desiccation resistance is helpful for insects that are frequently exposed to dehydrating environments, as is the case of most beetles thriving in arid regions (Zachariassen, 1991; Le Lagadec et al., 1998), some studies have also questioned the factors that can constrain the development of desiccation resistance and the ecological costs this could have for the individual (Chown et al., 2011). Yet, there are few investigations that examined the eco-physiological responses of the insects exposed to desiccating conditions and that subsequently assessed the ecological costs over the long-term, by, for instance, measuring the reproductive output of the adults. With the unstoppable global warming which increases the frequency of drought events (IPCC, 2021), it has become crucial to improve our knowledge of the immediate effects of desiccating conditions on insects, as well as the cascading effects this can have on the long term. To fill this knowledge gap, we have used the tenebrionid beetle *A. diaperinus*, an insect of tropical origin whose ability to handle desiccating conditions has already been demonstrated (Renault and Coray, 2004; Renault et al., 2015).

The survival results confirmed the relatively high capacities of adult *A. diaperinus* to handle desiccating conditions, with the most resistant individuals being able to survive longer than 45 days without water at low relative humidity conditions (about 10% RH). Our findings are consistent with the former investigations performed by Renault and Coray (2004) who reported survival durations up to 30 days in adults of the species maintained at a higher temperature (29 ± 1°C) and lower humidity (5 ± 1% RH). Also, using a similar experimental design, Renault et al. (2015) reported an Lt_50_ of about 20 days for adult *A. diaperinus* exposed to desiccating conditions. As expected, mortality in our study was much higher in the desiccation treatment, where insects were additionally starved, as compared with the control group (which was also starved). In the latter group, the 25% of the beetles that had died after approximately 6 weeks of experiment has likely resulted from the exhaustion of body reserves of the individuals.

The initial body water content of adult *A. diaperinus* was around 55%, comparable to the 58.5% formerly reported by Salin et al. (1999), and to the body water content reported by Bjørge et al. (2018) which ranged from 58 to 67% depending on temperature and tested population of the species. Here, 4 weeks after the experiment had started, body water content of the beetles exposed to desiccating conditions was significantly lower than in control beetles. A similar pattern was reported from adults of *Paederus fuscipes* (Staphylinidae) whose body water amounts were quickly decreased after 2 weeks under desiccating conditions (Wang et al., 2019). Importantly, the body water loss pattern in individuals of *A. diaperinus* was consistent with survival results where we reported that mortality of the insects subjected to desiccating conditions started to increase more quickly after about 3 weeks. This finding may suggest that mortality started when body water content reached approximately 45% in the insect and corresponded to a percentage of total body water loss of about 46% at the time of death.

Earlier investigations demonstrated that tenebrionid beetles have a low cuticular permeability, and, together with the regulation of respiratory mechanism (Ahearn, 1970; Zachariassen, 1991), these mechanisms enhance their ability to maintain body water over prolonged exposures to dry conditions. Thus, we wanted to investigate if the activity of the insects would change significantly when they were exposed to a desiccating environment. Indeed, the initiation of a “sit-and-wait” strategy (Hervant and Renault, 2002) would greatly contribute to reducing aerobic metabolism, and thus the loss of metabolic water. Yet, when assessing the activity of the beetles, an increased locomotion was recorded in insects from control and desiccation treatments 2 weeks after the experiment had started. While this might be a surprising result at first, a deeper analysis of this response suggests that the transfer of the beetles to the Petri dish for activity assays may have stimulated exploration of this new environment, and more specifically favored foraging behavior (Lorenzo and Lazzari, 1998) with insects actively searching for food and water, and for a more suitable habitat (Yu et al., 2010). Consistently, adults of *A. diaperinus* that were maintained in the same environment for the whole desiccation assay remained mainly inactive (Renault and Coray, 2004). Renault et al. (2003) also found that adults of *A. diaperinus* starved for a week, but maintained at optimal relative humidity conditions, were characterized by an initial increase in activity which was depicted by a high increase in oxygen consumption, followed by a sit-and-wait strategy (Renault et al., 2003).

In several insects, the ability to survive dehydration is also associated with the accumulation of sugars (Nakahara et al., 2008). Meanwhile, in the present work, total soluble sugar content was significantly reduced in the beetles from both experimental treatments within the first week of the experiment, and this has likely resulted from the food deprivation that was imposed to the insects. Graves et al. (1992) showed in strains of *Drosophila* selected for desiccation resistance and having higher carbohydrate contents that stored sugars were depleted after stress exposure. Similarly, Renault et al. (2002) formerly found that ca. 17% of the carbohydrate stores (in the form of glycogen) were depleted in starved adult *A. diaperinus* ket at 24°C. The decreased amounts of circulating sugars we found may also reveal a lower energetic demand of the insects that were less active when coping with desiccating and starvation exposures.

The metabolic phenotypes of the insects from the two treatment groups followed a similar pattern over time. After 1 week, beetles from control and desiccation treatments were both different from the remaining experimental time points, indicating that food deprivation had a more pronounced effect on the insects’ metabolic phenotype than desiccation. Consistent with the measurements of circulating sugars, levels of trehalose, which is the main energetic sugar in insects (Thompson, 2003) fall after 1 week, and subsequently remained at low amounts, including in insects from the desiccating condition. This compound has been repeatedly reported as an actively accumulated metabolite in a range of arthropod species facing low relative humidity conditions (Benoit et al., 2009; Thorat et al., 2012), but did not appear as a major actor of responses to desiccation in adult *A. diaperinus*. Osmolarity, calculated by summing the quantities of metabolites measured by GC-MS, also remained constant throughout the experimental period for both treatment groups. Altogether, our results suggest that adult *A. diaperinus* have developed mechanisms aiming at limiting the rate of body water loss rather than increasing tolerance to water loss. In other words, it seems that the species is desiccation resistant rather than desiccation tolerant. However, it is also important to keep in mind that the reduction in body water incurred by the desiccation treatment has probably contributed to increasing the concentration of the metabolites in the body fluids of the beetles. Yet to avoid repetition, the resulting increased concentration of metabolites in the insects’ body water did not represent the physiological response we were expecting (accumulation of compatible solutes in the body fluids). To our knowledge, the osmolarity of metabolites has not been investigated for tenebrionid beetles previously, but total osmolarity and ions were measured in the tenebrionid *Stips stali*, and the authors found that the species had increased osmolality through sodium, potassium, and chloride concentration augmentation during dehydration (Naidu and Hattingh, 1986). In future studies, it would be thus interesting to measure the ion contribution to osmolarity in adult *A. diaperinus* exposed to desiccating conditions.

As the adults are exhibiting high desiccation resistance, as depicted by their prolonged survival durations at low RH conditions, we were hypothesizing that this would have subsequent costs for the fitness of the insects, in particular in terms of reproductive capacities. Our findings evidenced the impact an 8 day exposure to desiccation subsequently had on the number of larvae produced by the adults. It seems that exposure to desiccating conditions had flow-on effects on gametes and or egg laying. Since all experimental insects were fed during the 8 d treatment prior to assaying reproductive abilities, the reduction in energy stores may have been limited, and the results rather reflected the impact of desiccation only. Reduced ovarian maturation and number of mature oocytes in the ovaries have been shown in the Olive fruit fly (Diptera: Tephritidae) maintained at low relative humidity (12 and 33% RH) (Broufas et al., 2009). Furthermore, cessation of reproduction after exposure to dry conditions has been shown in Collembola (Waagner et al., 2011), and arrest in egg laying under desiccating conditions has also been demonstrated in several insect species (Vessby, 2001; Pappas et al., 2008; Norhisham et al., 2013). It would be interesting to examine if the reproductive capacity of adult *A. diaperinus* could return to similar values to that of the control insects after some time to better assess the long-term nature of the observed effects. Considering that effects on eggs and larvae are often more severe than in adults, this study performed on adults indicates that desiccating conditions can have detrimental effects on population size of *A. diaperinus* (Urs and Hopkins, 1973; Holmstrup, 2019).

## Conclusion

We have found that adults of *A. diaperinus* are highly resistant to desiccation, which can explain its success as a pest in very dry habitats. Interestingly, the exposure to desiccating conditions was not accompanied by physiological responses in the form of accumulation of compatible osmolytes (sugars, polyols, amino acids). We suggest that future studies examine changes in the concentration of inorganic solutes (for instance Na^+^, K^+^, Cl^–^), changes in the expression of Late Embryonic Abundant proteins (LEA) and aquaporins, all of these factors being potential modulators of the level of desiccation resistance in the lesser mealworm. Despite high desiccation tolerance, the number of offspring recorded from adults after they were exposed to desiccating conditions for 1 week was significantly reduced, proving the physiological costs stress exposure had on the individuals.

## Data Availability Statement

The original contributions presented in the study are included in the article/Supplementary Material; further inquiries can be directed to the corresponding author/s.

## Author Contributions

DR executed the experiments and ran the laboratory analyses. JED analyzed the data. DR and JED wrote the first draft of the manuscript and revised the manuscript. Both authors contributed to the article and approved the submitted version.

## Conflict of Interest

The authors declare that the research was conducted in the absence of any commercial or financial relationships that could be construed as a potential conflict of interest.

## Publisher’s Note

All claims expressed in this article are solely those of the authors and do not necessarily represent those of their affiliated organizations, or those of the publisher, the editors and the reviewers. Any product that may be evaluated in this article, or claim that may be made by its manufacturer, is not guaranteed or endorsed by the publisher.

## Footnotes

1
www.metaboanalyst.ca
