# Supplementary material for: Ecophysiological Responses of the Lesser Mealworm Alphitobius diaperinus Exposed to Desiccating Conditions
Source: Front Physiol. 2022 Feb 23;13:826458. doi: 10.3389/fphys.2022.826458 (PMC8905145; doi:10.3389/fphys.2022.826458)
Supplement: Supplementary file 1 [file Table_1.DOCX]

Appendix 1 - 1 Loadings plot of **(A)** component 1 (PC1) and **(B)** component 2 (PC2) from the PCA
