# Supplementary material for: Ecophysiological Responses of the Lesser Mealworm Alphitobius diaperinus Exposed to Desiccating Conditions
Source: Front Physiol. 2022 Feb 23;13:826458. doi: 10.3389/fphys.2022.826458 (PMC8905145; doi:10.3389/fphys.2022.826458)
Supplement: Supplementary file 2 [file Presentation_1.PPTX]

## Slide 1
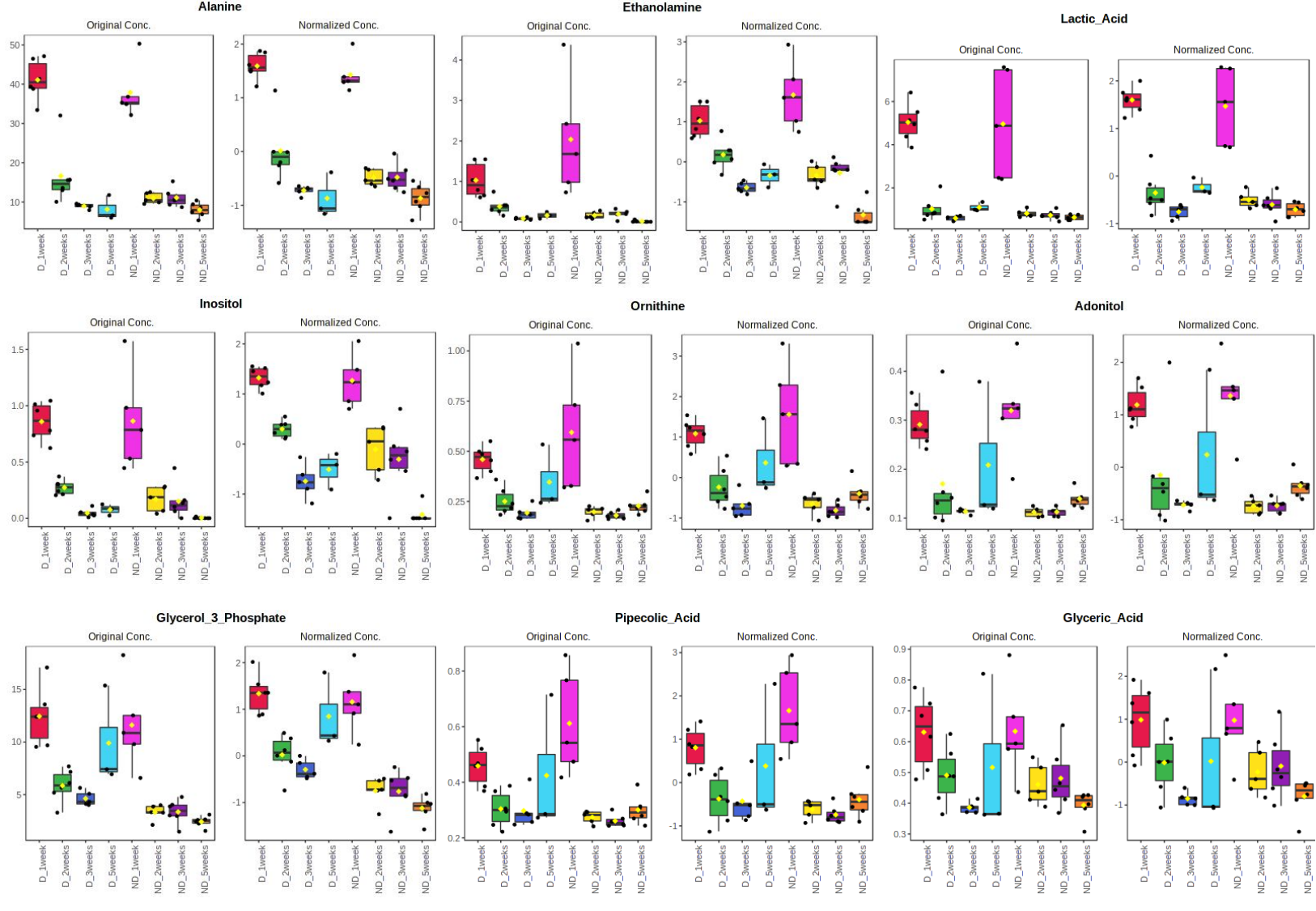

## Slide 2
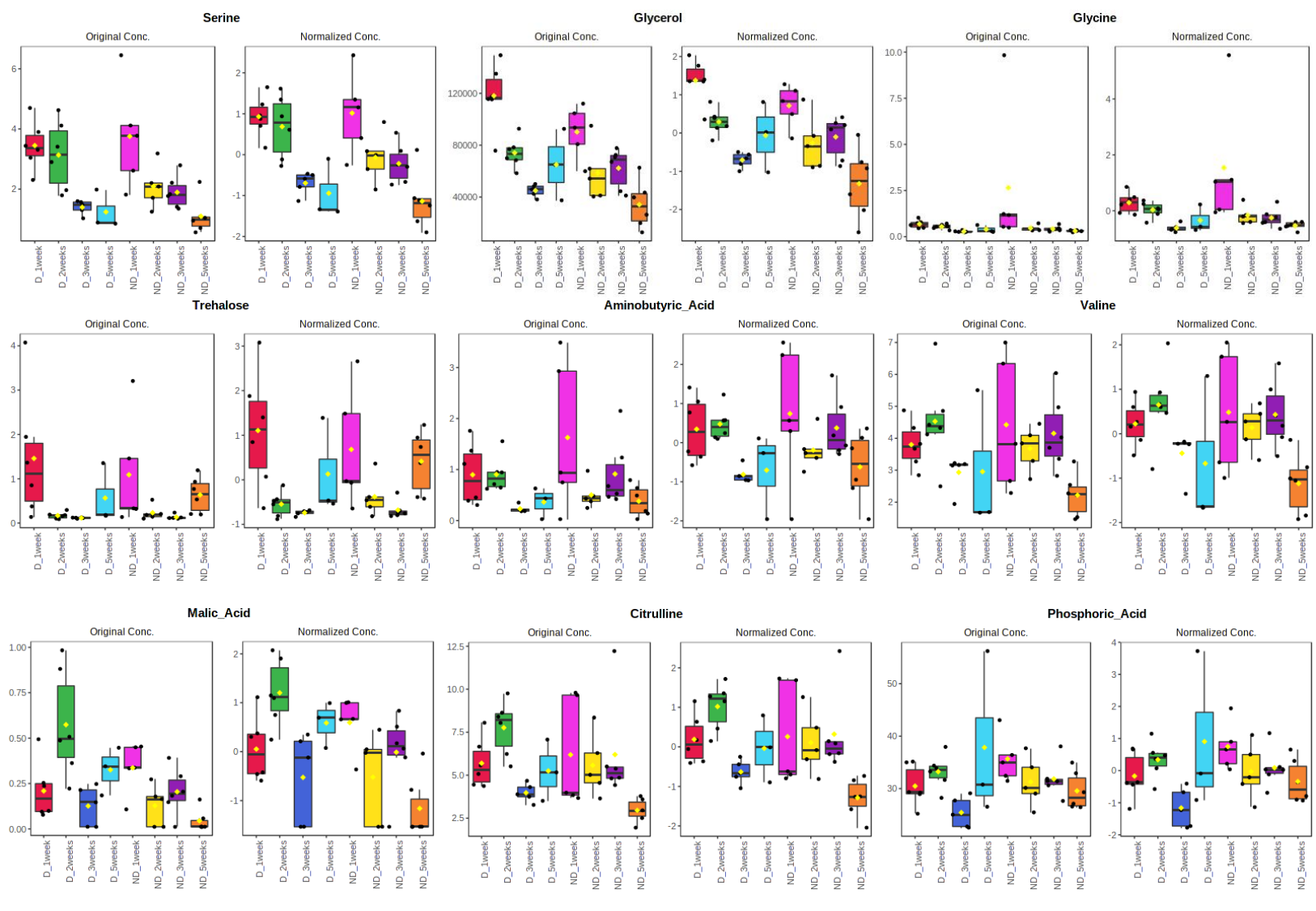

## Slide 3
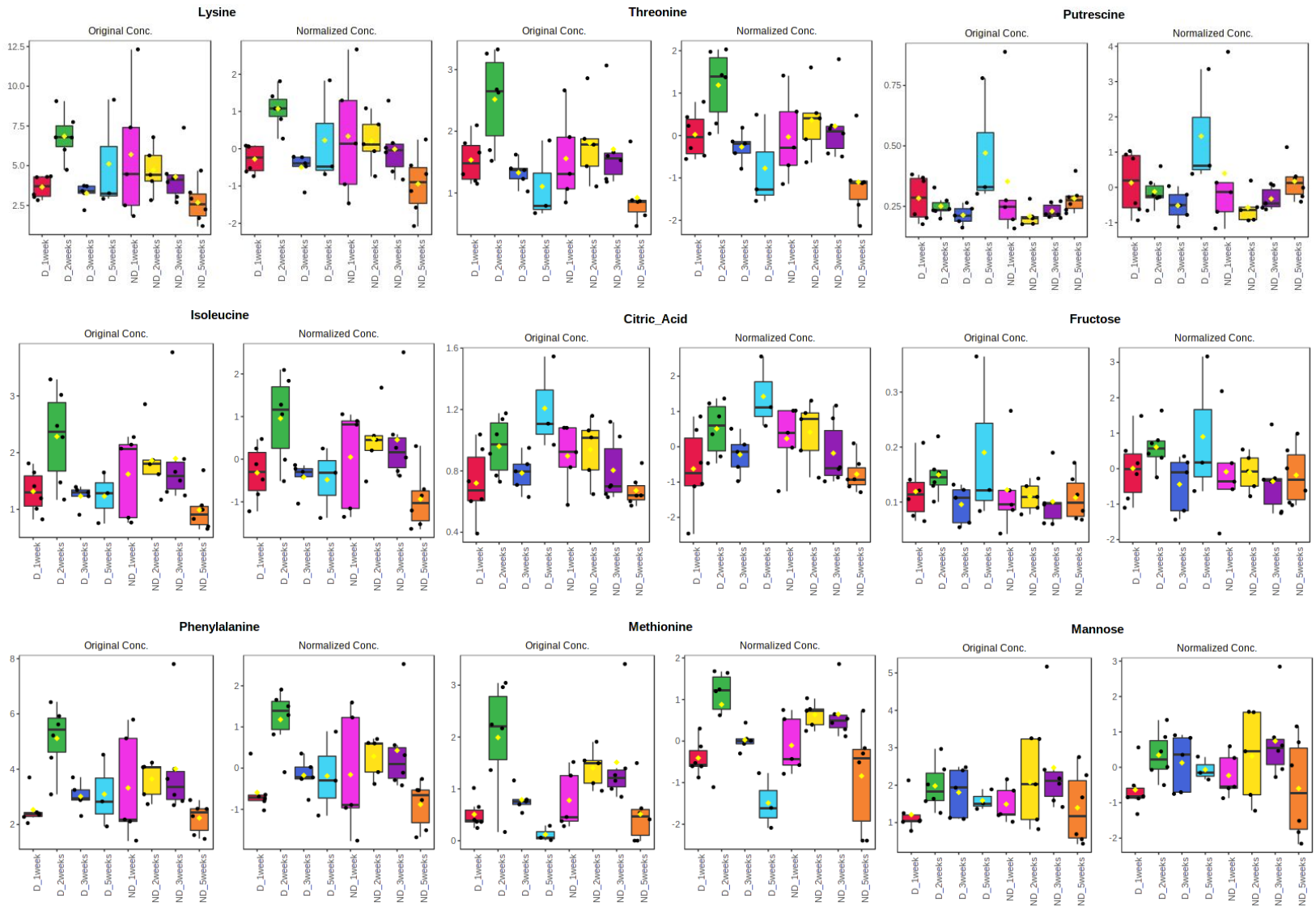

## Slide 4
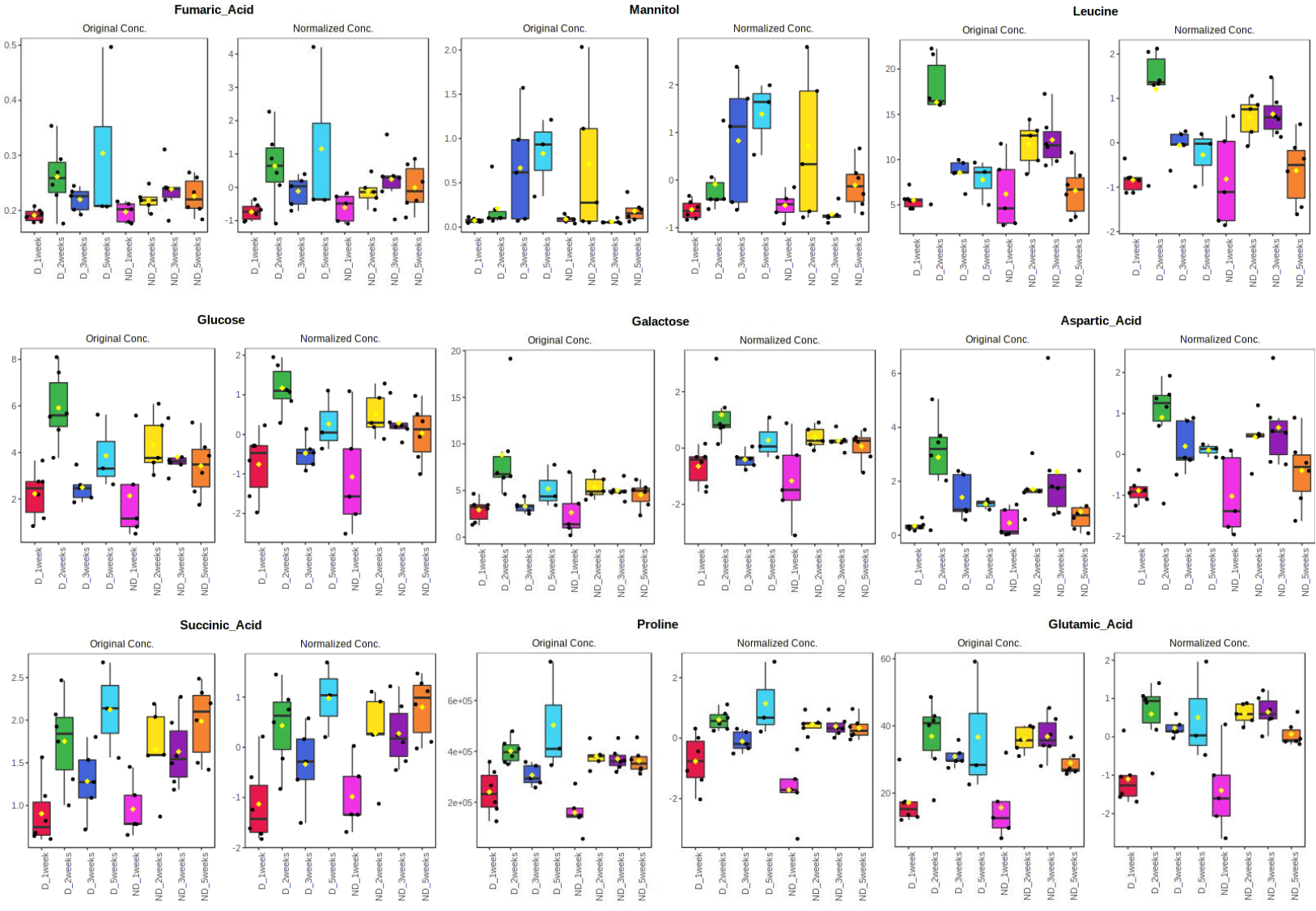

## Slide 5
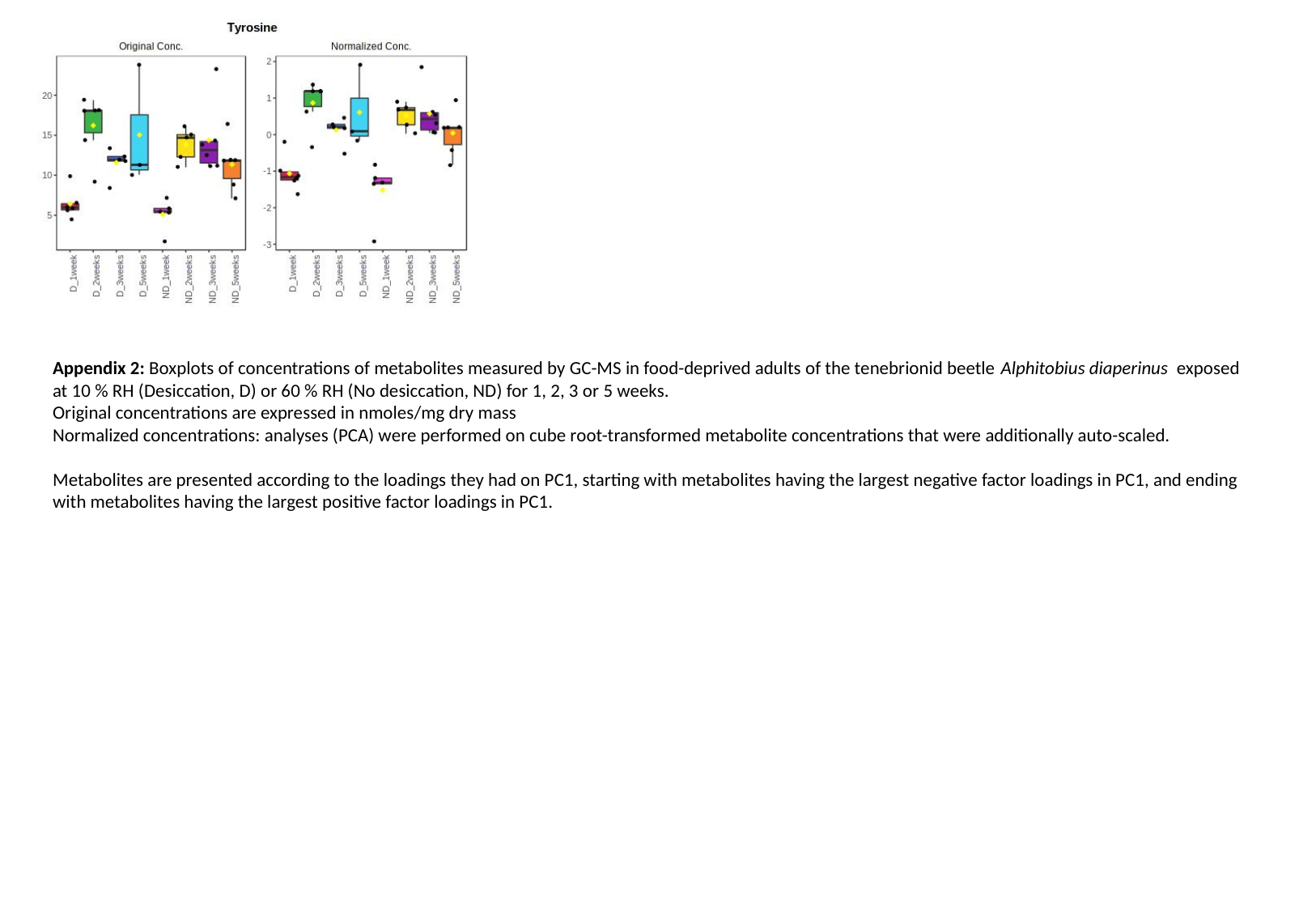

Appendix 2: Boxplots of concentrations of metabolites measured by GC-MS in food-deprived adults of the tenebrionid beetle Alphitobius diaperinus exposed
at 10 % RH (Desiccation, D) or 60 % RH (No desiccation, ND) for 1, 2, 3 or 5 weeks.
Original concentrations are expressed in nmoles/mg dry mass
Normalized concentrations: analyses (PCA) were performed on cube root-transformed metabolite concentrations that were additionally auto-scaled.
Metabolites are presented according to the loadings they had on PC1, starting with metabolites having the largest negative factor loadings in PC1, and ending
with metabolites having the largest positive factor loadings in PC1.
